# Sex Differences in rt-PA Utilization at Hospitals Treating Stroke: The National Inpatient Sample

**DOI:** 10.3389/fneur.2017.00500

**Published:** 2017-09-27

**Authors:** Amelia K. Boehme, Brendan G. Carr, Scott Eric Kasner, Karen C. Albright, Michael J. Kallan, Mitchell S. V. Elkind, Charles C. Branas, Michael T. Mullen

**Affiliations:** ^1^Department of Neurology, Mailman School of Public Health, College of Physicians and Surgeons, Columbia University, New York, NY, United States; ^2^Department of Epidemiology, Mailman School of Public Health, Columbia University, New York, NY, United States; ^3^Department of Epidemiology, School of Public Health, Birmingham, AL, United States; ^4^Department of Emergency Medicine, Thomas Jefferson University, Philadelphia, PA, United States; ^5^Department of Neurology, University of Pennsylvania, Philadelphia, PA, United States; ^6^Geriatric Research Education and Clinical Center (GRECC), Birmingham VA Medical Center, Birmingham, AL, United States; ^7^Department of Neurology, School of Medicine, University of Alabama at Birmingham, Birmingham, AL, United States; ^8^Center for Clinical Epidemiology and Biostatistics, University of Pennsylvania, Philadelphia, PA, United States; ^9^Leonard Davis Health Institute, University of Pennsylvania, Philadelphia, PA, United States

**Keywords:** acute stroke care, thrombolysis, health policy, emergency care, healthcare delivery systems

## Abstract

**Background and purpose:**

Sex and race disparities in recombinant tissue plasminogen activator (rt-PA) use have been reported. We sought to explore sex and race differences in the utilization of rt-PA at primary stroke centers (PSCs) compared to non-PSCs across the US.

**Methods:**

Data from the National (Nationwide) Inpatient Sample (NIS) 2004–2010 was utilized to assess sex differences in treatment for ischemic stroke in PSCs compared to non-PSCs.

**Results:**

There were 304,152 hospitalizations with a primary diagnosis of ischemic stroke between 2004 and 2010 in the analysis: 75,160 (24.7%) patients were evaluated at a PSC. A little over half of the patients evaluated at PSCs were female (53.8%). A lower proportion of women than men received rt-PA at both PSCs (6.8 vs. 7.5%, *p* < 0.001) and non-PSCs (2.3 vs. 2.8%, *p* < 0.001). After adjustment for potential confounders the odds of being treated with rt-PA remained lower for women regardless of presentation to a PSC (OR 0.87, 95% CI 0.81–0.94) or non-PSC (OR 0.88, 95% CI 0.82–0.94). After stratifying by sex and race, the lowest absolute treatment rates were observed in black women (4.4% at PSC, 1.9% at non-PSC). The odds of treatment, relative to white men, was however lowest for white women (PSC OR = 0.85, 95% CI 0.78–0.93; non-PSC OR = 0.80, 95% CI 0.75–0.85). In the multivariable model, sex did not modify the effect of PSC certification on rt-PA utilization (*p*-value for interaction = 0.58).

**Conclusion:**

Women are less likely to receive rt-PA than men at both PSCs and non-PSCs. Absolute treatment rates are lowest in black women, although the relative difference in men and women was greatest for white women.

## Introduction

Intravenous recombinant tissue plasminogen activator (IV rt-PA) is a thrombolytic agent that improves outcomes in stroke patients when administered within 4.5 h of symptom onset (1–3). The utilization of this therapy in the United States is low despite consensus guidelines recommending its use (4, 5). Although patient-level factors, primarily a delay from symptom onset to emergency department arrival, are a major contributor to the low utilization of IV rt-PA, provider- and hospital-level factors may also contribute to underutilization. The Joint Commission (TJC) primary stroke center (PSC) certification, which requires an acute stroke team, a stroke unit, and written care protocols, has been associated with higher rt-PA utilization and lower mortality among patients when compared to patients treated at non-PSCs (6–10).

Sex disparities in IV rt-PA use have been reported; however, whether PSCs reduce these disparities is unknown (11–13). Given this, an analysis of IV rt-PA treatment comparing non-PSCs and PSCs across the US would fill a gap in our understanding. When compared with men, women have worse functional outcomes and lower quality of life after stroke (14, 15). While this may be attributable to patient-level factors, such as older age at time of stroke or differences in stroke subtype, there is also evidence that women receive lower quality of care than men, with less efficient stroke evaluation, delay in treatment with thrombolytics, and lower adherence to stroke care quality metrics (16–18).

Further contributing to the sex disparity in IV rt-PA utilization is evidence that sex differences may vary by race/ethnicity, with black women being treated less frequently than white women (19). Building on prior work evaluating differences in rt-PA by race/ethnicity (20), we used the National (Nationwide) Inpatient Sample (NIS), to explore differences in the utilization of rt-PA at PSCs compared to non-PSCs, stratified by both sex and race/ethnicity.

## Materials and Methods

### Study Design

A retrospective, cross-sectional analysis of data from the NIS from 2004 to 2010 was conducted. As part of the Agency for Healthcare Research and Quality’s Healthcare Cost and Utilization Project, the NIS is the largest publicly available all-payer inpatient care database (21). This analysis limits the data to 26 states that publicly identified both treating hospital and patient race/ethnicity (Table S1 in Supplementary Material). Data on PSC certification and date of initial certification for PSCs was obtained *via* personal communication from TJC on May 17th, 2011 (Jean Range, executive director of disease specific care, TJC, unpublished data, 2011). Methods for this work have been previously reported (7, 20).

We identified patients aged ≥18 years who had a primary diagnosis of ischemic stroke defined by *International Classification of Diseases, 9th Revision* (ICD-9) codes 433.x1, 434.x1, and 436. The positive predictive value of these codes for identifying acute ischemic stroke is greater than 85% (22). We excluded patients transferred from other hospitals. Patients who were missing information on death, sex, length of stay, or primary payer were excluded. Treatment with IV rt-PA, defined by ICD-9 procedure code 99.10, was the primary outcome. When compared to pharmacy billing data, this code has been shown to identify 77% of IV rt-PA cases (23). This study involved the analysis of de-identified data and was exempt from IRB approval.

### Demographic Variables

The NIS classifies sex as male or female. Race/ethnicity is reported as non-Hispanic white, non-Hispanic black, Hispanic, Asian/Pacific Islander, Native American, or other. Race and ethnicity are not reported separately. Other patient-level variables included age, year of discharge, expected primary payer (Medicaid, Medicare, private, other), median household income in the patient’s ZIP code, comorbid conditions (Table S2 in Supplementary Material) (24), and an all-patient refined diagnosis related group (APR-DRG) measure of the expected risk of inpatient mortality. The APR-DRG uses diagnosis and procedure codes to estimate the likelihood of dying during the hospitalization as minor, moderate, major, or extreme (25). The APR-DRG marker is not specific to stroke and does not include a measure of stroke severity. Hospital-level variables included geographic region (Northeast, Midwest, South, West), rural or urban location, status as a teaching hospital (yes/no), and annual ischemic stroke case volume (<100, 100–299, ≥300).

### Statistical Analysis

The statistical approach for this study parallels prior work from our group using the NIS to evaluate rt-PA use. Baseline characteristics were described for patients treated at PSCs and non-PSCs using measures of central tendency (means, medians) for continuous variables and proportions for categorical variables. Differences between the groups were evaluated using Student’s *t*, Wilcoxon rank-sum, and χ^2^ tests, as appropriate. Patients were stratified by sex, and then further stratified by race/ethnicity within sex. A multivariate model was constructed to determine independent associations including year of discharge, age, sex, primary expected payer, median income by ZIP code, hospital region, teaching status, urban/rural location, and ischemic stroke admission volume, the 29 Elixhauser comorbid conditions, and the APR-DRG measure of disease severity (20). Our analytic models used NIS survey statistics and Taylor series estimation to account for the survey design and clustering within hospitals. The analysis was conducted using SAS-callable-SUDAAN version 11.0.1. As this was an exploratory analysis, no adjustments were made for multiple comparisons (26). An alpha of 0.05 was set as the level of significance.

## Results

Between 2004 and 2010, acute ischemic stroke was the primary diagnosis for 598,606 hospitalizations in the NIS. The inclusion/exclusion criteria for the study population and the baseline patient- and hospital-level characteristics stratified by PSC status have been previously published (20). Table 1 describes the patient- and hospital-level characteristics for the sample, stratified by sex. Overall, 53.4% of the patients were women. Among women, 71.9% were white, 15.5% black, 7.4% Hispanic, and 5.2% were in other categories (e.g., Asian/Pacific Islander, Native American, other). Among men, 71.0% were white, 14.4% were black, 8.6% Hispanic, and 6.0% other. There were 75,160 (24.7%) patients evaluated at a PSC, and 228,992 (75.3%) evaluated at a non-PSC.

**Table 1:** Patient and hospital characteristics stratified by sex.

	Overall patient population	Male patients *n* = 141,841	Female patients *n* = 162,311
			
	*n* = 304,152	(46.6%)	(53.4%)
	%	%	%
Evaluated at a primary stroke center^a^	24.7	25.4	24.1
**Race/ethnicity^a^**			
White	71.5	71.0	71.9
Black	15.0	14.4	15.5
Hispanic	7.9	8.6	7.4
Other^b^	5.6	6.0	5.2
**Age^a^**
18–44	3.9	4.3	3.6
45–64	25.1	31.4	19.6
65–80	37.7	40.0	35.7
>80	33.3	24.3	41.1
**Income^a^^,^^c^**
Lowest quartile	23.5	23.0	23.8
Second quartile	24.2	24.1	24.3
Third quartile	24.1	24.2	24.1
Highest quartile	26.0	26.1	25.8
Missing	2.3	2.6	2.0
**Payment typea**
Medicare	68.0	61.9	73.3
Medicaid	6.8	6.9	6.7
Private, incl. HMO	18.9	22.9	15.4
Self-pay	3.7	4.9	2.7
No charge	0.6	0.7	0.4
Other	2.0	2.6	1.5
**Hospital regiond**
Northeast	30.9	30.6	31.1
Midwest	10.2	9.9	10.4
South	32.4	32.8	32.1
West	26.5	26.7	26.4
Rural hospital location^e^	10.5	10.2	10.8
Teaching hospital^a^	41.1	42.1	40.3
**Ischemic stroke volumea**
<100 (cases/year)	14.5	13.9	15.1
100–299 (cases/year)	46.6	46.2	46.9
≥300 (cases/year)	38.9	40.0	38.1

*^a^χ^2^ test between males and females significant, p < 0.001*.

*^b^Other includes Asian/Pacific Islander, Native American, or other*.

*^c^Median household income, by ZIP code*.

*^d^χ^2^ test between males and females significant, p = 0.003*.

*^e^χ^2^ test between males and females significant, p = 0.001*.

**Figure 1:**
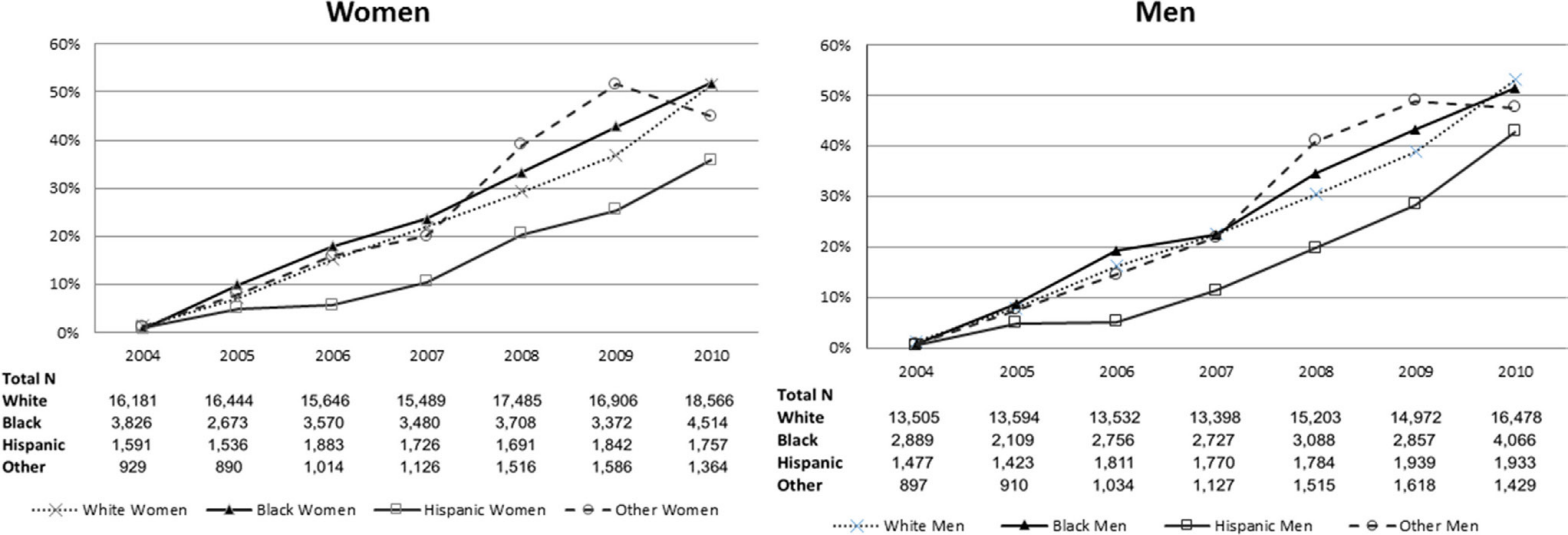
Proportion of patients presenting to a primary stroke center over time, stratified by sex and then by race/ethnicity.

The proportion of patients evaluated at a PSC increased over time with similar proportions for men and women. The proportion of racial/ethnicity groups presenting to a PSC stratified by sex increased over time (Figure 1). A little over half of the patients evaluated at PSCs were women (53.8%), with 71.5% of white, 14.5% of black, 8.8% of Hispanic, and 5.3% of other patients evaluated at a PSC.

**Figure 2:**
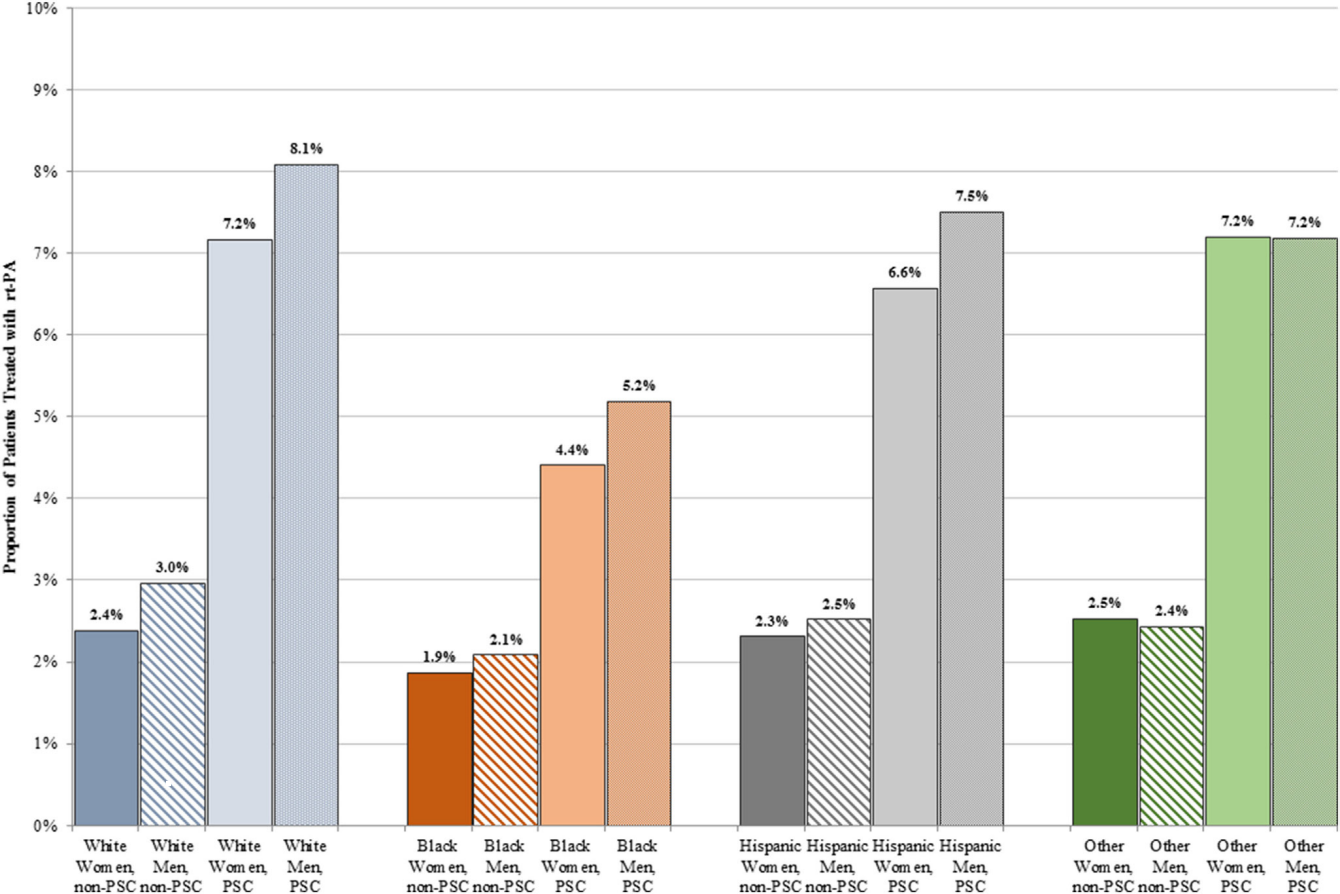
Proportion of men and women treated with rt-PA stratified by race/ethnicity and primary stroke center (PSC) versus non-PSC.

A lower proportion of women than men received rt-PA at both PSCs (6.8 vs. 7.5%, *p* < 0.001) and non-PSCs (2.3 vs. 2.8%, *p* < 0.001). Figure 2 shows treatment rates stratified by sex and race. A higher proportion of patients received rt-PA at PSCs in all sex and race/ethnicity groups. The proportion of rt-PA treated patients at PSCs ranged from 4.4% in black women to 8.1% in white men. The proportion of rt-PA treated patients at non-PSCs ranged from 1.9% for black women to 3.0% for white men.

In an unadjusted model, women were at lower odds of being treated with rt-tPA compared to men at both PSCs and non-PSCs (Table 2). After adjusting for year, age, primary expected payer, median income quartiles by ZIP code, region, teaching hospital, urban hospital location, annual ischemic stroke case volume, 29 AHRQ individual comorbidities, and APR-DRG risk of mortality, the odds of being treated with rt-PA remained significantly lower for women regardless of presentation to a PSC (OR 0.87, 95% CI 0.81–0.94) or non-PSC (OR 0.88, 95% CI 0.0.82–0.94). After stratifying by race, white women had significantly lower odds of rt-tPA treatment in the adjusted model (Table 2). In the multivariable model, sex did not modify the effect of PSC status on rt-PA utilization (*p*-value for interaction = 0.58).

**Table 2:** Odds of being treated with rt-PA for women, relative to men, at non-PSCs and primary stroke centers (PSCs), stratified by race/ethnicity.

Sex	Non-PSC		PSC	
	Unadjusted OR (95% CI)	Adjusted OR (95% CI)	Unadjusted OR (95% CI)	Adjusted OR (95% CI)
Overall	0.83 (0.78–0.88)	0.88 (0.82–0.94)	0.88 (0.83–0.93)	0.87 (0.81–0.94)
**By race/ethnicity**				
White	0.80 (0.75–0.85)	0.84 (0.78–0.90)	0.88 (0.82–0.94)	0.85 (0.78–0.93)
Black	0.90 (0.78–1.04)	0.99 (0.82–1.20)	0.84 (0.72–0.99)	0.96 (0.77–1.21)
Hispanic	0.92 (0.74–1.12)	1.04 (0.82–1.33)	0.87 (0.66–1.14)	0.85 (0.61–1.18)
Other	1.04 (0.80–1.35)	1.09 (0.77–1.53)	1.00 (0.81–1.24)	0.91 (0.67–1.25)

*Adjusted for (where applicable): year, age, sex, primary expected payer, median income quartiles by ZIP, region, teaching hospital, urban hospital location, annual ischemic stroke case volume, 29 AHRQ individual comorbidities, all-patient refined diagnosis related group risk of mortality (minor, moderate, major, and extreme likelihoods of dying)*.

## Discussion

Our results illustrate that the relationship between PSC certification and rt-PA utilization is similar in men and women. PSCs utilize rt-PA more than non-PSCs in both men and women, regardless of race/ethnicity. However, our results also illustrate that women are less likely to receive rt-PA than men at both PSCs and non-PSCs. In absolute treatment proportions, black patients are treated in lower proportions than other racial/ethnicity groups, and black women are treated less than any other group; however, the relative odds of treatment were lowest in white women relative to white men.

Sex disparities in the treatment of stroke remain a concern, particularly with the rise in incident stroke among women. IV rt-PA is effective for both men and women, but women may be even more likely to benefit from treatment than men (27, 28). Among patients not treated with rt-PA, women may have worse outcomes than men (29). It is therefore critical to ensure that women with stroke who are eligible for rt-PA receive treatment. PSCs administer more rt-PA than non-PSCs. This difference is equally true in men and in women. As more PSCs become certified throughout the US and as a greater proportion of stroke patients are evaluated at PSCs, IV rt-PA treatment in women should continue to increase. However, a better understanding of the drivers of sex differences in rt-PA treatment is needed to maximize the population benefit of developing systems of care and eliminate existing disparities. Identification of these drivers is of particular importance considering the evidence that rt-PA is associated with a greater improvement in stroke symptoms for women than for men, with no differences in adverse events after rt-PA administration between men and women (30, 31).

Lower rt-PA treatment rates in women may be related to patient factors. First, women tend to be older than men at the time of stroke. Second, women tend to present with more severe strokes (32). Third, knowledge of the signs and symptoms of stroke may be lower in women (14). This could potentially contribute to a delay in emergency department presentation, thereby preventing women from being treated if presentation is outside of the treatment time window (33). Unfortunately, the National Inpatient Sample does not contain the granular clinical data necessary to investigate these possibilities. More research is needed to determine why women are treated at a lower proportion than men so that sex disparities in acute stroke therapies can be eliminated.

Black women have the lowest proportion of rt-PA treatment, regardless of PSC status of the treating hospital. This finding supports prior data from two academic medical centers, which also found that black women were treated less frequently with rt-PA. In prior studies, black women were more likely to have delayed presentation, and once time from onset was accounted for, the sex and race disparity in rt-PA treatment no longer remained (19). It is possible that differences in time to presentation are responsible for the findings in the present study, but as noted above, symptom duration is not available in the NIS.

This study has limitations similar to prior work evaluating rt-PA use in the NIS (7, 20). As this study relies on administrative claims data defined by ICD-9 codes, we were unable to determine if there were differences in rt-PA eligibility, stroke severity, or time to presentation. We do not have information on functional outcomes after rt-PA in this dataset and cannot assess sex differences in outcomes. States that do not provide data on hospital identity or race were excluded, including a few with large minority populations, such as Georgia and Texas. In spite of this, the 26 states that provided data cover approximately 50% of the Hispanic and black population in the United States (34, 35). The definition of PSCs by TJC certification does not recognize other national or state-based certifications or identify hospitals that participate in national stroke care improvement programs, such as Get with the Guidelines. Misclassification of these hospitals as non-stroke centers would likely bias the results toward the null and would not change the observed disparities. The use of the ICD-9 procedure code 99.10 to define rt-PA may underestimate rt-PA use. This has the potential to introduce bias if coding varies between PSCs and non-PSCs or, less likely, between women and men (36). Finally, we are unable to investigate “drip and ship” cases. This could lead to underestimation of rt-PA utilization at non-PSCs.

## Conclusion

The sex disparities seen in rt-PA utilization remain after accounting for race/ethnicity and other known factors that contribute to rt-PA underutilization. The increased utilization of rt-PA at PSCs is consistent between men and women and stable across racial and ethnic groups. Considering women may benefit from rt-PA utilization more than men, the disparity in utilization is concerning. Further research is needed to investigate what factors are contributing to this sex disparity.

## Ethics Statement

This study was the analysis of de-identified data from the National Inpatient Sample and was exempt from IRB approval.

## Author Contributions

AB is the primary author of this manuscript. BC and SK made critical revisions to the manuscript. KA contributed to the study idea, data interpretation, and made critical revisions. MK conducted the data analyses and made critical revisions. ME and CB made critical revisions. MM developed the study idea, obtained the data, interpreted the data, and made critical revisions.

## Conflict of Interest Statement

BC spends a portion of his time in the Office of the Assistant Secretary for Preparedness and Response. The findings/conclusions of this report are those of the author and do not necessarily represent the views of the Department of Health and Human Services or its components. The other authors have no conflicts to report.
