# Supplementary material for: Sex Differences in rt-PA Utilization at Hospitals Treating Stroke: The National Inpatient Sample
Source: Front Neurol. 2017 Sep 27;8:500. doi: 10.3389/fneur.2017.00500 (PMC5623663; doi:10.3389/fneur.2017.00500)
Supplement: Supplementary file 1 [file Table_1.DOCX]

ONLINE SUPPLEMENT

Title: **Sex Disparities in IV rt-PA Use Persist at Primary Stroke Centers**

**SUPPLEMENTAL TABLES:**

Table I. States with Identifiable Hospitals and Race/Ethnicity in the NIS, 2004-2010

| Year | Total # States | # States w/ Identifiable Hospitals and Race | States Reporting  Hospital Identity and Race |
| --- | --- | --- | --- |
| 2004 | 37 | 18 | AZ, CA, CO, CT, FL, IA, MD, MA, MO, NH, NJ, NY, NC, RI, UT, VT, VA, WI |
|  |  |  |  |
| 2005 | 37 | 17 | AZ, CA, CO, CT, FL, IA, MD, MA, MO, NH, NJ, NY, NC, RI, UT, VT, WI |
|  |  |  |  |
| 2006 | 38 | 18 | AZ, CA, CO, CT, FL, IA, MD, MA, MO, NH, NJ, NY, NC, RI, UT, VT, VA, WI, |
|  |  |  |  |
| 2007 | 40 | 18 | AZ, CA, CO, CT, FL, IA, MD, MA, MO, NH, NJ, NY, NC, RI, UT, VT, VA, WI |
|  |  |  |  |
| 2008 | 42 | 23 | AZ, CA, CO, CT, FL, IA, KY, MD, MA, MO, NV, NH, NJ, NY, NC, OR, PA, RI, UT, VT, VA, WA, WI |
|  |  |  |  |
| 2009 | 44 | 23 | AZ, CA, CO, CT, FL, IL, IA, KY, MD, MA, MT, NV, NH, NJ, NY, OR, PA, RI, UT, VT, VA, WA, WI |
|  |  |  |  |
| 2010 | 45 | 23 | AZ, CA, CO, CT, FL, IL, IA, KY, MD, MA, MS, MT, NV, NJ, NY, NC, OR, PA, RI, UT, VT, VA, WI |
|  |  |  |  |

Table II. Elixhauser Comorbidities

| AIDS |
| --- |
| Alcohol Abuse |
| (Deficiency) Anemias |
| Rheumatoid Arthritis |
| Blood Loss Anemia |
| Congestive Heart Failure |
| Chronic Pulmonary Disease |
| Coagulopathy |
| Depression |
| Diabetes (Uncomplicated) |
| Diabetes (w/ Chronic Complications) |
| Drug Abuse |
| Hypertension |
| Hypothyroidism |
| Liver Disease |
| Lymphoma |
| Fluid and Electrolyte Disorders |
| Metastatic Cancer |
| Other Neurological Disorders |
| Obesity |
| Paralysis |
| Peripheral Vascular Disorders |
| Psychoses |
| Pulmonary Circulation Disorders |
| Renal Failure |
| Solid Tumor (without Metastasis) |
| Peptic Ulcer Disease (no bleeding) |
| Valvular Disease |
| Weight Loss |
